# Detection of vertebrates from natural and artificial inland water bodies in a semi‐arid habitat using eDNA from filtered, swept, and sediment samples

**DOI:** 10.1002/ece3.10014

**Published:** 2023-04-24

**Authors:** Rupert McDonald, Philip W. Bateman, Christine Cooper, Mieke van der Heyde, Mahsa Mousavi‐Derazmahalleh, Brock A. Hedges, Michelle T. Guzik, Paul Nevill

**Affiliations:** ^1^ Trace and Environmental DNA Laboratory, School of Life and Molecular Sciences Curtin University Perth Australia; ^2^ Behavioural Ecology Lab, School of Molecular and Life Sciences Curtin University Perth Australia; ^3^ School of Molecular and Life Sciences Curtin University Perth Australia; ^4^ School of Biological Sciences The University of Adelaide Adelaide Australia

**Keywords:** arid lands, biodiversity, biomonitoring, environmental DNA, Fauna surveys, metabarcoding, vertebrates

## Abstract

Biomonitoring is vital for establishing baseline data that is needed to identify and quantify ecological change and to inform management and conservation activities. However, biomonitoring and biodiversity assessment in arid environments, which are predicted to cover 56% of the Earth's land surface by 2100, can be prohibitively time consuming, expensive, and logistically challenging due to their often remote and inhospitable nature. Sampling of environmental DNA (eDNA) coupled with high‐throughput sequencing is an emerging biodiversity assessment method. Here we explore the application of eDNA metabarcoding and various sampling approaches to estimate vertebrate richness and assemblage at human‐constructed and natural water sources in a semi‐arid region of Western Australia. Three sampling methods: sediment samples, filtering through a membrane with a pump, and membrane sweeping in the water body, were compared using two eDNA metabarcoding assays, 12S‐V5 and 16smam, for 120 eDNA samples collected from four gnammas (*gnamma*: Australian Indigenous Noongar language term–granite rock pools) and four cattle troughs in the Great Western Woodlands, Western Australia. We detected higher vertebrate richness in samples from cattle troughs and found differences between assemblages detected in gnammas (more birds and amphibians) and cattle troughs (more mammals, including feral taxa). Total vertebrate richness was not different between swept and filtered samples, but all sampling methods yielded different assemblages. Our findings indicate that eDNA surveys in arid lands will benefit from collecting multiple samples at multiple water sources to avoid underestimating vertebrate richness. The high concentration of eDNA in small, isolated water bodies permits the use of sweep sampling that simplifies sample collection, processing, and storage, particularly when assessing vertebrate biodiversity across large spatial scales.

## Graphical abstract

Our findings indicate that eDNA surveys in arid lands will benefit from collecting multiple samples at multiple water sources to avoid underestimating vertebrate richness. The high concentration of eDNA in small, isolated water bodies permits the use of sweep sampling which simplifies sample collection, processing, and storage, particularly when assessing vertebrate biodiversity across large spatial scales.

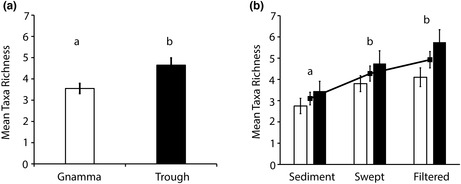

## 1 INTRODUCTION

Semi‐arid and arid lands comprise more than one third of the Earth's land surface and include ecosystems such as forests, woodlands, savannas, shrublands, grasslands, and deserts (Lemons & Victor, 2003). Despite limited water availability due to a combination of low rainfall, high temperatures, and low humidity, these environments harbor a remarkably diverse biota, high levels of endemism and some of the world's most endangered species (Durant et al., 2012). Semi‐arid and arid ecosystems have received less attention than other ecosystems, irrespective of their extent, ecological and social value, and the threats they face (Durant et al., 2012; Razgour et al., 2018). The extent of arid habitats is increasing globally due to climate change, and it is predicted they will cover 56% of the Earth's land surface by 2100 (Lemons & Victor, 2003; O'Farrell et al., 2010). Habitat degradation is a major issue for existing semi‐arid and arid lands (Lemons & Victor, 2003; O'Farrell et al., 2010) and poses a significant threat to ecosystem function and services, upon which the livelihoods of more than 250 million people rely (James et al., 2013).

Biomonitoring is vital for establishing baseline data that is needed to identify and quantify ecological change and to inform management and conservation activities in semi‐arid, arid, and other habitats (Campbell et al., 2002; Epanchin‐Niell et al., 2012; Herrick et al., 2006). Conventional techniques such as observational surveys (direct observation, camera traps, audio recordings), recording field signs (hair/feathers, scats, tracks, feeding signs) and live trapping can be costly, labor intensive and may have ethical considerations (e.g., Cross et al., 2020; Furlan et al., 2020; Waudby et al., 2019). Some species may also have lower detectability than others, leading to biases in survey data (e.g., Einoder et al., 2018; Fernandes et al., 2018; Ji et al., 2013; Thompson & Newmaster, 2014). As a result, conventional monitoring techniques have some limitations, particularly detecting species of high priority for conservation or management, which are often rare, endangered, invasive, or elusive (Barnes & Turner, 2016; Harper, Handley, et al., 2019; Kelly et al., 2014; Rodgers & Mock, 2015). Therefore, there is an urgent need for biomonitoring practices that can generate detailed and current environmental information on arid ecosystems (Kelly et al., 2014).

Environmental DNA (eDNA) metabarcoding is a relatively new biomonitoring tool that has potential to overcome some of the disadvantages of more conventional approaches (Barnes & Turner, 2016). Metabarcoding involves sequencing small regions of DNA isolated from substrate samples and comparing the sequences against a library of taxonomically identified sequences to determine the species present in the sample (Taberlet et al., 2012). High‐throughput sequencing has revolutionized the application of eDNA, enabling ecological communities to be characterized at a relatively low cost due to the simultaneous analysis of many samples (Furlan et al., 2020; Kelly et al., 2014). Metabarcoding of eDNA has proved highly successful in freshwater and marine systems (Egeter et al., 2018; Furlan et al., 2020; Harper, Handley, et al., 2019; Palacios et al., 2020; Thomsen et al., 2012; Ushio et al., 2017, Wang et al., 2021). However, despite rapid advancements in the technology, it is currently difficult to apply metabarcoding to terrestrial biomonitoring where eDNA may not be well preserved (van der Heyde et al., 2020), appropriate sampling substrates may be limited (Fahner et al., 2016; van der Heyde et al., 2020, 2021), consistent protocols to sample substrates are not well established (Harrison et al., 2019; van der Heyde et al., 2022), and reference databases for arid habitat taxa are incomplete (Bradford et al., 2010; Carrasco‐Puga et al., 2021; Egeter et al., 2018; Palacios et al., 2020; van der Heyde et al., 2020). As a result, the application of eDNA for biomonitoring in arid lands is rare.

In water‐limited environments, both natural and human‐constructed (artificial) water bodies are critical water sources for animals (Davies, 1972; Hedges et al., 2021; James et al., 1999; Redfern et al., 2003). Consequently, these water bodies may be particularly rich sources of eDNA and important for biodiversity monitoring as they can provide a broad snapshot of biodiversity over a wide area given that animals may migrate over vast distances to water(. Studies have shown the applicability of eDNA metabarcoding in identifying fauna using water holes (Farrell et al., 2022; Furlan et al., 2020; Harper, Handley, et al., 2019; Mas‐Carrió et al., 2021) but, to date, no studies have explored the use of different sampling methods (e.g., filtering vs sweeping vs sediment). Sample volume protocols designed for marine environments (i.e., 1–5 L) are typically applied to freshwater habitats (Egeter et al., 2018; Palacios et al., 2020; Takahashi et al., 2023) despite distinct differences in water characteristics. Water bodies in semi‐arid and arid lands are often of a relatively small volume and a greater turbidity (due to high concentrations of algae, sediment, and organic debris) than marine or other freshwater environments (Harper, Buxton, et al., 2019). Attempts to apply sampling protocols for marine environments to studies of water bodies in semi‐arid and arid habitats have been hindered by pump blockages during filtering of water for analysis, reducing the number of samples processed (Egeter et al., 2018; Klymus et al., 2017). Techniques to reduce sample turbidity (e.g., centrifugation, increased filter membrane pore size, pre‐filtering) may reduce eDNA capture, especially when eDNA is bound to suspended particles (Turner et al., 2014), and increase costs and contamination risk (Klymus et al., 2017; Takahara et al., 2012). Turbidity issues can be avoided by collecting sediments rather than water, with the additional benefits of reduced eDNA decay in sediment compared to water (Buxton et al., 2018; Turner et al., 2015) and a high yield of eDNA due to binding of DNA to particulate matter (Turner et al., 2015). However, there is evidence that humic substances in sediment inhibit PCR amplification (e.g., Stoeckle et al., 2017) and that differences in eDNA viability between substrates can lead to a failure to detect all the components of the faunal community (Palacios et al., 2020).

Biomonitoring using eDNA metabarcoding has been used previously to detect terrestrial vertebrates in artificial water sources (Rodgers & Mock, 2015), but no studies to date have compared detection of eDNA from natural and artificial water sources in the same ecosystem. Here, we sampled water from both natural and artificial water bodies. While both water bodies are accessed by numerous fauna, there are likely to be differences in the community composition between them due to the location, structure, size, and immediate surroundings (e.g., Korine et al., 2016; Letnic et al., 2014; Schneider & Griesser, 2009). Here we tested the hypothesis that vertebrate eDNA can be detected in water and sediment samples collected from both gnammas (natural water sources) and cattle troughs (artificial water sources) in the semi‐arid Great Western Woodlands, Western Australia. We investigated whether the vertebrate taxonomic richness and community assemblages detected with eDNA metabarcoding techniques differed for natural and artificial water sources and examined the effect of sampling methodology on these variables. We compared results from (1) sediment samples, (2) water samples filtered through membranes (filtered samples) and (3) unfiltered samples collected on membranes dipped in the water (swept samples; Bessey et al., 2021) and evaluated the effect of sample replication on detected taxonomic richness. Our overall aim was to expand the scope of eDNA metabarcoding to biomonitoring in environments previously considered suboptimal for the application of eDNA approaches, thereby enhancing the potential of eDNA techniques for conservation and management.

## 2 MATERIALS AND METHODS

### 2.1 Study sites

Fifteen samples were collected from each of four gnammas and four cattle troughs (*n* = 120; see below), located in a 96 km^2^ area of the Fraser Range, 380 km NNE of Esperance, Western Australia. The study site has a semi‐arid climate with 268 mm of annual rainfall occurring year‐round. Vegetation communities are dominated by *Eucalyptus* woodlands and *Acacia* shrublands and herb lands. The Fraser Range is within the Great Western Woodlands (GWW), the world's largest semi‐arid to arid woodland (Newbey et al., 1984), which covers 16 million hectares and supports an exceptional native flora (>3300 plant species) and fauna (49 native mammal, 11 feral mammal, 138 reptile, 14 frog, and 215 bird species; Department of Parks and Wildlife, 2013; Fox et al., 2016).

### 2.2 Sample collection

Water samples, sediment samples, and samples from membranes swept through the water body were collected in May 2021 (late Autumn) from the four gnammas and four cattle troughs. Five replicate samples of each type were taken from each water source. Gnammas ranged from 30 cm to 300 cm in width, and 20 cm to 100 cm in depth. Cattle troughs were 130 cm wide and 60 cm deep. Five 50 mL water samples were collected into falcon tubes at five random locations in each water source, at a depth of 5–15 cm from the surface. Five Supor 47 mm 0.45 μm pore‐size filter membranes (Pall Corporation) were submerged at a depth of 5–15 cm at five random locations from each water source, and “swished” around for 15 s before being placed immediately into individual zip‐lock bags. Finally, five randomly chosen sediment samples were scooped from the bottom of each water source into a 50 mL falcon tube. Disposable gloves were worn during sampling and were changed between every sample at each location. Samples were placed on ice immediately after collection. Water samples (50 mL) were filtered within 24 h through a Supor 47 mm 0.45 μm pore size filter membrane (Pall Corporation) using a Sentino Microbiology Pump (Pall Corporation). Equipment was decontaminated between samples in a 10% bleach solution for 10 min and rinsed thoroughly before subsequent use. Two filtering control samples were taken by pumping 500 mL of local tap water through filter membranes. All samples were kept on ice from the time of collection or processing and were frozen at −20°C at Curtin University within 72 h of collection.

### 2.3 Sample processing and DNA extraction

Prior to DNA extraction, samples were defrosted in a refrigerator (4°C) overnight. DNA was extracted from filter membranes using the Qiagen DNeasy Blood and Tissue Kit (Qiagen). Filter membranes were cut up into 2 mm wide strips and incubated in 540 mL buffer ATL and 60 μL proteinase K at 56°C for 3 h. Sediment samples were homogenized using a TissueLyser (Qiagen) and DNA was extracted from 250 mg of sediment using the DNeasy PowerLyser Powersoil Kit (Qiagen), which contains steps to remove inhibitors from the extracts. Samples were then extracted using the QIAcube automated platform (Qiagen) and eluted to 100 μL using the manufacturer's protocol. DNA extracts were immediately frozen at −20°C. DNA extraction blanks (negative controls) were processed with each batch of 30 samples (*n* = 4) using the extraction reagents only.

### 2.4 Assessment of DNA extracts

Due to the degraded nature of eDNA, metabarcoding primers typically target short barcode regions to improve amplification success (Yu et al., 2012). The primers used were the 12Sv5‐F/R targeting the mitochondrial 12S gene (F: 5'‐TAGAACAGGCTCCTCTAG‐3'; R: 5'‐TTAGATACCCCACTATGC‐3', ~98 bp (Riaz et al., 2011) and the mammal specific primers 16Smam1/2 targeted the mitochondrial 16S ribosomal gene (F: 5'‐CGGTTGGGGTGACCTCGGA‐3'; R: 5'‐GCTGTTATCCCTAGGGTAACT‐3', ~135 bp (Taylor, 1996). Both assays target regions conserved across vertebrates (12S‐V5) or mammals (16Smamm) and can be recovered from degraded DNA (Kitano et al., 2007; Sarri et al., 2014; Staats et al., 2016). Quantitative polymerase chain reaction (qPCR) was used to detect the quality and quantity of DNA in each extract, and extract and verify the optimum DNA input for metabarcoding (Murray et al., 2015). Here, qPCR assays were run on all samples using the 12S‐V5 F/R primers on the neat extract, 1/5 and 1/10 dilution to screen for PCR inhibitors in the reaction. The extraction of sediment samples included inhibitor removal steps because those tend to be more abundant in sediments, but all sample types were screened. The majority of water/passive samples were found to have an optimum dilution in the realm of neat, 1/5 or 1/10, whereas the majority of sediment samples had to be diluted to 1/100. Using the optimum dilutions determined by qPCR with 12S‐V5 primers, a supplementary qPCR assay was run on a subset of 20 samples to determine whether the 16smam forward/reverse primers were as effective at amplifying DNA at these dilutions. In some instances, primer bias can differentially amplify eDNA at sites with different community composition (Aird et al., 2011). To mitigate these effects the combination of a vertebrate primer and a specific mammal primer were used. In addition, 12S and 16S are broadly used for mammal detection in eDNA metabarcoding studies and there is a greater availability of reference sequences available online for taxonomic assignment (Deagle et al., 2014; Valentini et al., 2016).

The polymerase chain reaction (PCR) mix for quantification contained: 2.5 mM MgCl2 (Applied Biosystems), 10 × PCR Gold buffer (Applied Biosystems), 0.25 mM dNTPs (Astral Scientific), 0.4 mg/mL bovine serum albumin (Fisher Biotec), 0.4 μmol/L forward and reverse primer, 1 U AmpliTaq Gold DNA polymerase (Applied Biosystems), and 0.6 μL of a 1:10,000 solution of SYBR Green dye (Life Technologies). All PCR amplification was conducted on a StepOne Plus (Applied BioSystems) real‐time qPCR instrument with the following conditions: 5 min at 95°C, 50 cycles of 95°C for 30 s, 30 s at the annealing temperature (58°C) and 45 s at 72°C, followed by 15 s at 95°C, 1 min at 60°C, and 15 s at 95°C during the melt curve stage, ending with 10 min of elongation at 72°C. Contamination was minimized by preparing the PCR mixes in a dedicated clean laboratory, and then adding DNA extract in a separate laboratory, inside specialized ultraviolet hoods.

### 2.5 DNA amplification and sequencing

Based on qPCR results, fusion tagging was performed on samples that contained adequate amplifiable DNA by assigning each sample a unique combination of fusion tag primers. Each fusion tag primer combination contained a unique multiplex identifier between 6 and 8 bp in length, Illumina's sequencing adaptors (i.e., P5 and P7) and the gene‐specific primer (described above). A single‐step fusion protocol was carried out with unique index combinations before qPCR was used to generate amplicons of each fusion‐tagged sample using the same reagents and cycling conditions as described above. The fusion‐tagged amplicons were generated in duplicates for each biological replicate to maximize amplicon numbers for sequencing and reduce the chances of non‐detections and the effects of PCR stochasticity (Murray et al., 2015). PCR replicates were then pooled, amplicons cleaned using the QIAquick PCR Purification Kit (Qiagen) and then quantified using the QIAxcel Advanced System (Qiagen). Based on this quantification, the DNA library for sequencing was made from pools, combined in approximate equal concentrations. A Pippin Prep (Sage Science) was used to size‐select the amplicons in this library, and the library was then cleaned using the QIAquick PCR Purification Kit (Qiagen). A Qubit fluorometer (Thermo Fisher Scientific) was used to quantify the final DNA library, before sequencing as per Illumina's sequencing protocols for single‐end sequencing, using Illumina's single direction MiSeq 300 V2 Reagent Kit on the Illumina MiSeq platform (Illumina).

### 2.6 Sequence analysis and taxonomic assignment

Using a high‐performance computing cluster (Pawsey Supercomputing Centre), sequences were analyzed with the eDNAFlow automated pipeline (Mousavi‐Derazmahalleh et al., 2021), which performed the following tasks: sequence quality was checked with FASTQC (Andrews, 2010) and filtered with AdapterRemoval v2 (Schubert et al., 2016) for Phred quality score lower than 20 and trimming sequences with Ns as enforced in eDNAFlow by—trimns and—trimqualities parameters. Remaining trimmed sequences were demultiplexed and sequences smaller than expected minimum amplicon length were trimmed (12S‐V5 minimum length 50 bp; 16smam minimum length 25 bp) using OBITools' ngsfilter and obigrep tools, respectively (Boyer et al., 2016). Unique sequences, zero‐radius operational taxonomic units (ZOTUs‐denoised sequences) and an abundance table were generated using the USEARCH (Edgar, 2010) commands fastx‐uniques, unoise3, (minsize 8) and otutab, respectively. The ZOTUs generated from both assays were queried against the nucleotide database Genbank (https://www.ncbi.nlm.nih.gov/genbank/) in October 2021 using the following parameters in Basic Local Alignment Search Tool (BLASTN) (Altschul et al., 1990): perc_identity ≥90, evalue ≤1e−3, best_hit_score_edge 0.05, best_hit_overhang 0.25, qcov_hsp_perc 100, max_target_seqs = 10. Taxonomic identification was then assigned with more strict parameters using the eDNAFlow Lowest Common Ancestor (LCA) script (Mousavi‐Derazmahalleh et al., 2021) with a minimum percentage identity (%identity) of ≥95, and if a ZOTU had multiple blast assignments where the difference between their %identity was equal to or smaller than 1, then that ZOTU was assigned to the nearest common taxonomic level otherwise a species level assignment was returned.

The results of the LCA script were compared against existing taxonomic data for the sampling area (Department of Parks and Wildlife, 2013), and if necessary, manually curated to ensure that the taxa assigned are known to occur in the sampling area. While the majority of ZOTUs were assigned to species level, the LCA script dropped a few ZOTUs to the nearest common taxonomic level (e.g., Meliphagidae *sp*.). Where ZOTUs were assigned to taxa that are not local to the sampling area, the ZOTUs were either reassigned to sister species, for example, black‐flanked rock‐wallaby (*Petrogale lateralis*) and Australian shelduck (*Tadorna tadornoides*) known to occur in the area, or dropped to the nearest common taxonomic level that currently exists in the sampling area, for example, vesper bats, (Vespertilionidae *sp*.), that is, ZOTUs were manually assigned to locally known species if all other species for that ZOTU were exotic to the sampling area: Australian magpie (*Gymnorhina tibicen*), yellow‐throated miner (*Manorina flavigula*), Australian magpie‐lark (*Grallina cyanoleuca*), euro (*Osphranter robustus*), and sheep (*Ovis aries*). In cases where ZOTUs were assigned to family level and multiple exotic species were attributed to a ZOTU with 100% identity, the ZOTUs were reassigned to generic rank of species known to be native to the sampling area (e.g., *Corvus* sp.). All ZOTUs assigned to “chordata environmental samples” were removed from the data set, and all ZOTUs assigned to exotic canid spp. (i.e., *Nyctereutes viverrinus*, *Canis lupus rufus*, *Canis lupus*) were reassigned to *Canis lupus familiaris*. ZOTUs assigned to exotic “Artiodactyla spp.” (i.e., *Bison bonasus*, *Bos javanicus*, *Bos mutus*, *Cephalophus dorsalis*, *Muntiacus* sp., *Pudu puda*, *Syncerus caffer*, *Tragelaphus eurycerus*) were reassigned to domestic cow (*Bos taurus*).

### 2.7 Statistical analysis

We generated a presence–absence matrix for the two assays (12S‐V5 and 16smam) for both sources (gnammas and cattle troughs) and all sampling methods (swept, filtered, and sediments). This matrix was used to calculate taxon richness for each sample. The effect of source and sampling method was evaluated using a two‐way analysis of variance (ANOVA) achieved using StatistiXL v 2.0 (www.statistiXL.com). The presence–absence matrix was also used to examine differences in biological assemblages identified from the various sources and sampling methods, using the PERMANOVA+ software add‐on in PRIMER7 (Clarke & Gorley, 2015). To avoid unassigned resemblance values, two gnamma‐sediment samples (GS1_3 and GS2_4) and one gnamma‐filter sample (GW2_2) for which no taxa were detected were removed from the matrix, before a Jaccard distance matrix was generated between samples. Using these data, a PERMANOVA was run using Type III sums of squares, unrestricted permutation of raw data and significance determined by 9999 permutations of the pseudo‐F statistic (Clarke & Gorley, 2015). PRIMER7 was used to visualize and estimate pair‐wise tests to determine how the sources and sampling methods compared using non‐metric multidimensional scaling (nMDS).

We used the “BiodiversityR” (Kindt & Coe, 2005) and “drc” packages (Knezevic et al., 2007) with R 3.5.1 (R Core Team, 2018) to generate accumulation curves for taxa at each site and with each sampling method. Asymptotic regression rarefaction curves were generated for each sampling method and models were visualized using the package “ggplot2” (Chiarucci et al., 2008; Wickham & Sievert, 2016). Curves represent the order‐free accumulation of mean taxa detections calculated from random permutations of all possible orderings of taxa detections. We then used an EcoTest (Cayuela et al., 2015) to statistically compare rarefaction curves representing each sampling method for each source respectively, testing the null hypothesis that three samples were drawn from a single assemblage and any differences in their rarefaction curves reflect only sampling effects. Using the package “rareNMtests” (Cayuela et al., 2015) the abundances of all taxa detections were summed to generate a pooled composite curve. The test statistic (Z) was calculated from the cumulative summed areas of difference between the three individual curves representing each sampling method and the composite curve. The observed value of this Z‐statistic was compared to a null model distribution constructed from rarefaction curves generated from 200 random permutations of all possible orderings of taxa detections in each set of replicates across the three sampling methods and significance assessed at *α* = 0.05. A “leave one out” analysis was used to subsequently determine how z‐values and significance changed when sampling methods were individually omitted from the analysis, after Cayuela et al. (2015).

## 3 RESULTS

There was successful eDNA amplification from 110 and 111 of 120 samples using the 12S‐V5 and 16smam assays, respectively. Sequencing yielded 9,740,203 (mean per sample = 74,924 ± 4395 standard error; SE) sequences for the 12S‐V5 assay and 12,530,593 (mean per sample = 96,389 ± 5762 SE) for the 16smam assay sequences in total. A small proportion of sequence reads were present in the field and extraction controls, highest in the 16smam assay (3.09% of total reads) and lowest in the 12S‐V5 (2% of total reads). A proportion of sequence reads were assigned to humans, which were highest in the 16smam assay (27.05%) and lowest in the 12S‐V5 assay (13.84%); ZOTUs representing humans were not included in the statistical analysis. One ZOTU assigned to *Gallus* amplified with the 12S‐V5 assay accounted for 0.7% of total reads for that assay and was removed as a likely contaminant as *G. gallus* DNA is used in the laboratory as a positive control.

### 3.1 Vertebrate taxon richness detected across sources and sampling methods

**FIGURE 1:**
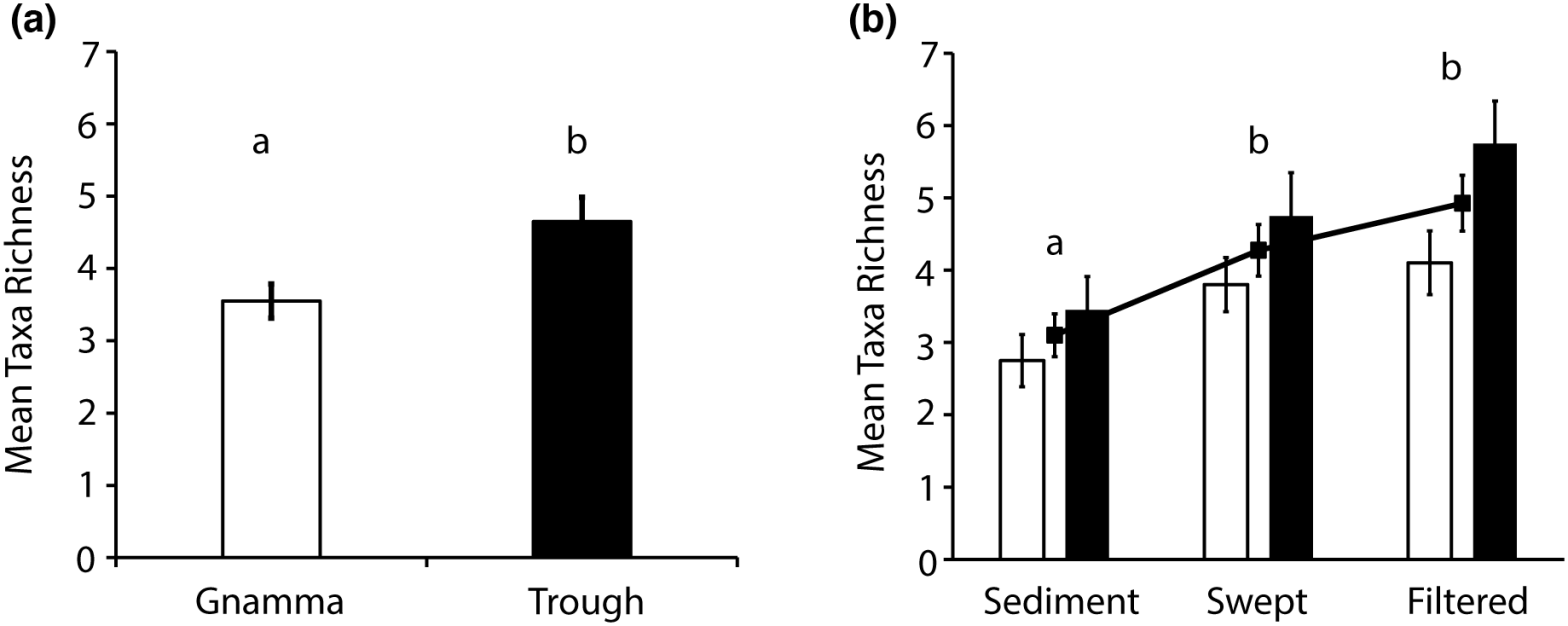
Vertebrate taxon richness detected in (a) gnammas (rock holes) and cattle troughs, and (b) using sediment, sweeping, and filtering sampling methods from the two sources in the Great Western Woodland, Western Australia. Letters indicate significant differences (*α* = 0.05) between factors. Values are mean ± SE for *n* = 5 from each of four gnammas and four troughs.

**FIGURE 2:**
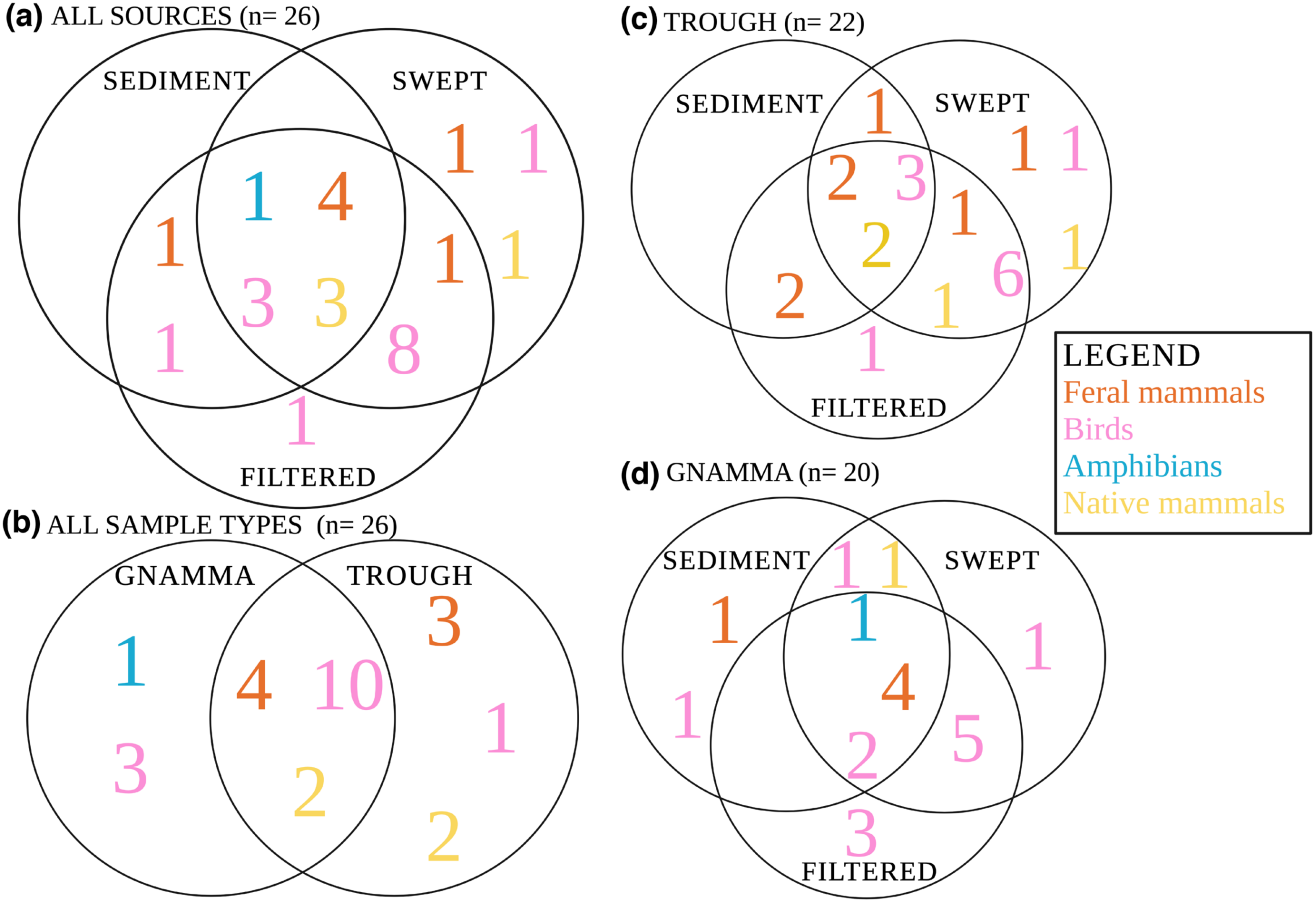
Tallies of feral mammals, birds, native mammals, and amphibians detected from sediments, swept water samples, and filtered water samples collected from gnamma holes (granite rock pools) and cattle troughs. Venn diagrams display the relationship of these tallies in (a) filtered (*n* = 22), swept (*n* = 23), and sediment (*n* = 13) samples from gnamma holes and cattle troughs, (b) gnamma holes (*n* = 20) and cattle troughs (*n* = 22) across all sampling methods (c) filtered (*n* = 18), swept (*n* = 19), and sediment (*n* = 10) samples collected from cattle troughs, (d) filtered (*n* = 15), swept (*n* = 15), and sediment samples (*n* = 11) collected from gnamma holes. Four gnammas and four troughs were sampled.

12S‐V5 and 16smam assays produced a total of 9190 and 3203 ZOTUs respectively, from which 6584 and 1144 were assigned. A total of 26 unique taxa were detected from both assays combined, representing 12 orders and 21 families including 11 mammal, 15 bird and 1 amphibian taxa (Table 1). The 12S‐V5 assay detected 25 unique taxa and the 16smam assay nine unique taxa. Eight common taxa were detected by both assays (Table 1). There were significant differences for total vertebrate richness between gnammas (3.55 ± 0.23 taxa per sample, 20 taxa total) and cattle troughs (4.65 ± 0.34 taxa per sample, 22 taxa total; *F*_5, 114_ = 7.9, *p* = .006) and between sampling methods (*F*_5, 114_ = 7.4, *p =* .001; Figure 1). The highest total richness was detected in swept (4.3 ± 0.36 taxa per sample; 23 taxa total) and filtered samples (4.9 ± 0.39 taxa per sample; 22 taxa total) while significantly fewer taxa were detected from sediment samples (3.1 ± 0.29 taxa per sample; 13 taxa total; *p ≤* .016; Figure 1 and Figure 2). No unique taxa were detected in sediment samples compared to water samples (both swept and filtered), while 13 taxa were only detected in water samples. Thirteen bird taxa were detected overall and nine were detected only in water samples and not in sediment. Similarly, 11 mammal taxa were detected and only three (*Ovis aries*, *Macropus fuliginosus* and *Osphranter robustus*) were detected in water and not in sediment (Figure 2).

**TABLE 1:** Vertebrate taxa detected from five samples each from four gnamma holes (granite rock pools) and four cattle troughs in the Great Western Woodlands, Western Australia, indicating sampling method (sediment, sweeping, and filtering) and assay (12S‐V5 and 16smam). Site indicates which of the gammas (G1–G4) and troughs (T1–T4) the taxa were detected in.

		Gnamma			Trough			Site		
		Sediment	Swept	Filtered	Sediment	Swept	Filtered	Sediment	Swept	Filtered
Amphibia	*Neobatrachus pelobatoides*	16S	16S	16S				G1/G3	G3	G1/G2/G3
Mammalia	*Macropus fuliginosus*					12S			T4	
	*Petrogale lateralis*				12S	12S	12S	T1	T1/T4	T1/T4
	*Osphranter robustus*	12S				12/16S		G2/G4		
									T1/T4	T1/T4
	*Oryctolagus cuniculus*				16S		16S	T4		T4
	*Vespertilionidae sp*.	12S	12S		12S	12S	12S	G3/G4	G2/G3	
								T1	T1/T4	T3
	*Felis catus*					12S			T1/T4	
	*Canis lupus familiaris*	12/16S	12/16S	12/16S	12/16S	12/16S	12/16S	G1/G2/G3/G4	G1/G2/G4	G1/G3/G4
								T1/T2/T3	T1/T2/T3/T4	T1/T2/T3/T4
	*Sus scrofa*	16S	12/16S	16S	12/16S	12S		G1	G1/G2/G3	G1/G2/G3/G4
								T1/T3/T4	T1	
	*Camelus dromedarius*	12/16S	16S	12/16S	16S		16S	G1/G2/G4	G1/G4	G1/G2/G3/G4
								T1/T2		T1/T4
	*Bos taurus*	12/16S	12/16S	12/16S	12/16S	12/16S	12/16S	G1/G2/G3/G4	G1/G2/G3/G4	G1/G2/G3/G4
								T1/T2/T3/T4	T1/T2/T3/T4	T1/T2/T3/T4
	*Ovis aries*					12S	12/16S		T4	T4
Aves	*Dromaius novaehollandiae*		12S	12S		12S	12S		G2/G4	G4
									T4	T2/T4
	*Falco peregrinus*		12S	12S					G2	G2
	*Tadorna tadornoides*			12S						G1
	*Eolophus roseicapilla*	12S		12S	12S	12S	12S	G1/G3/G4	G3/G4	
								T1/T2/T4	T1/T2/T3/T4	T1/T2/T3/T4
	*Northiella haematogaster*			12S		12S	12S			G1
									T1/T2/T4	T1/T2
	*Ocyphaps lophotes*	12S	12S	12S	12S	12S	12S	G1	G1	G1/G2/G4
								T1/T2/T3	T1/T2	T1/T2/T4
	*Phaps chalcoptera*	12S	12S	12S	12S	12S	12S	G1	G1/G2/G4	G1/G2/G3
								T1/T2/T3	T1/T2/T3/T4	T1/T2/T3/T4
	*Gymnorhina tibicen*		12S	12S		12S			G4	G4
									T2	
	*Manorina flavigula*			12S		12S	12S			G2/G3
									T2	T2
	*Pardalotus striatus*			12S					G2	
	*Meliphagidae sp*.		12S	12S		12S	12S		G3/G4	G1/G2/G3/G4
										T1/T2/T3
	*Corvus sp*.		12/16S	16S		12S	12S		G1/G3	G4
									T2	T2/T4
	*Grallina cyanoleuca*					12S	12S		T2	T2
	*Cinclosoma clarum*	12S					12S	G4		
										T3

### 3.2 Source and sampling effects on vertebrate taxon assemblages

There was a significant effect of source (*pseudo‐F =* 9.44; *p <* .001) and sampling method (*pseudo‐F =* 3.86; *p <* .001) on the vertebrate assemblages detected and a significant interaction between these factors (*pseudo‐F =* 2.32; *p =* .002;). All sampling techniques resulted in identification of different species assemblages for gnammas (*p* ≤ .013), but for cattle troughs the assemblages detected by swept samples did not differ from those detected by the filtered and sediment samples (*p* ≥ .050).

Differences between water sources appear to be driven by higher variation in amphibian and bird assemblages in gnamma samples and higher variation in mammal assemblages in cattle troughs (Table 1, Figure 2). For both sources there was a higher variation in the bird assemblage in both swept and filtered samples compared to sediment samples while the higher variation in the mammal assemblage in swept samples compared to filtered samples appeared to be driving the unique groupings observed in these sampling methods (Figure 2).

For gnammas, where the various treatments yielded statistically different species assemblages, a number of native birds were detected (i.e., *Manorina flavigula*, *Northiella haematogaster*, and *Tadorna tadornoides*) in filtered samples compared to swept samples where *Eolophus roseicapilla* (Australian galah) and a bat (Vespertilionidae sp.) were detected (Table 1). In contrast, *Osphranter robustus* and a few bird species (*Corvus* sp., *Cinclosoma clarum*, *Dromaius novaehollandiae*, *Falco peregrinus*, *Gymnorhina tibicen* and Meliphagidae sp.) were detected from sediment samples (Table 1). Meanwhile, eDNA metabarcoding of cattle trough filtered samples detected a few unique birds (*Corvus* sp., *D. novaehollandiae*, *Grallina cyanoleuca*, *Gymnorhina tibicen*, *Manorina flavigula*, *Northiella haematogaster* and Meliphagidae sp.) and mammals (*Felis catus*, *Ovis aries*, and *Osphranter robustus*) in contrast to sediment samples which yielded other mammals also detected in water samples (*Oryctolagus cuniculus* and *Camelus dromedarius*; Table 1).

### 3.3 Vertebrate taxa richness accumulation with replication effort

**FIGURE 3:**
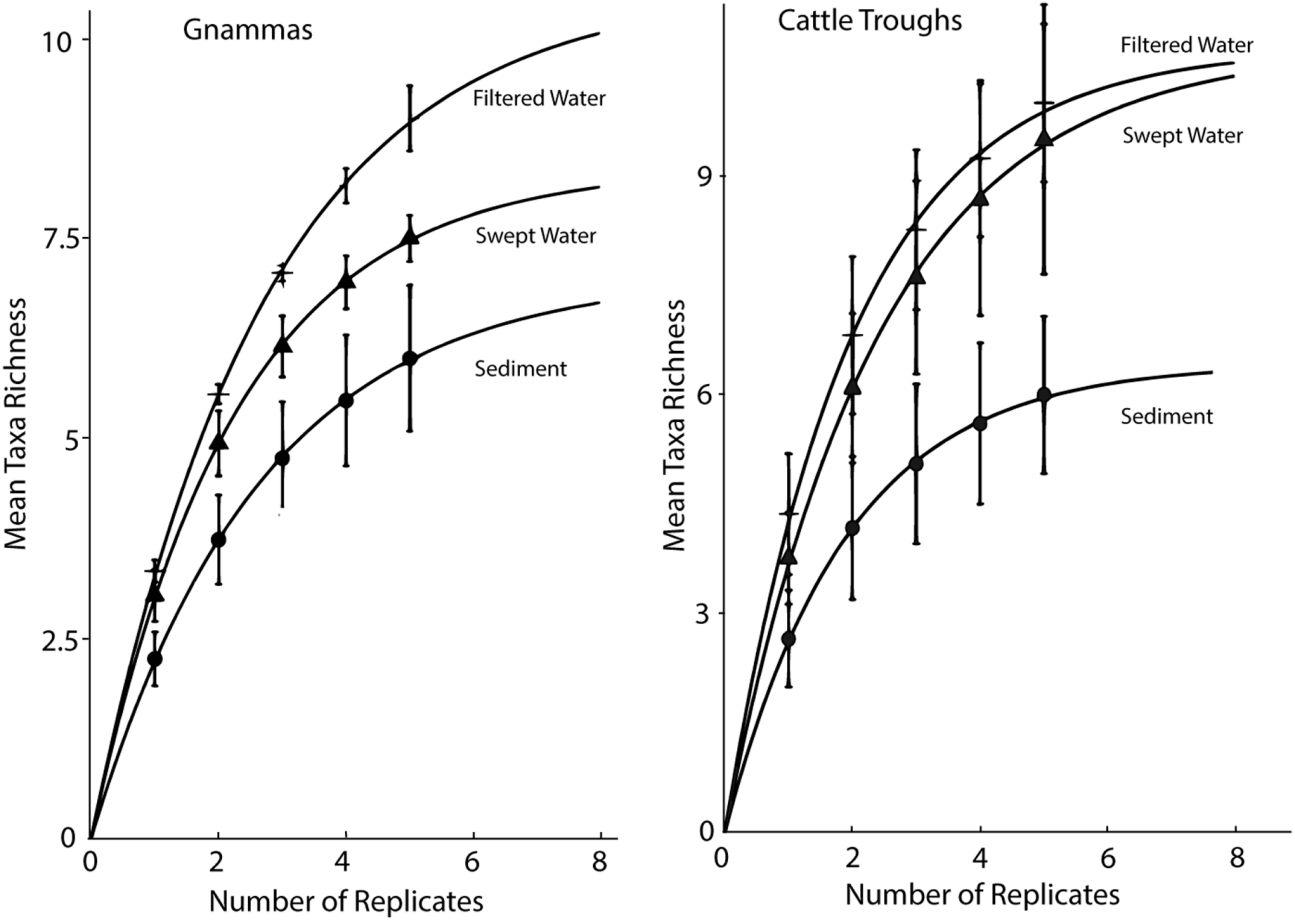
Rarefaction curves of vertebrate taxa detected with environmental DNA as a function of sampling effort in four gnammas and four cattle troughs in the Great Western Woodlands, Western Australia from samples collected from sediments, swept water samples, and filtered water samples.

Rarefaction EcoTests indicate that accumulation curves differed between sampling methods for both gnammas (*z* = 20.18; *p* = .040) and cattle troughs (*z* = 30.2; *p* = .045; Figure 3). However, when sediment was omitted from the analysis there was no difference between curves representing filter and sweeping sampling methods for either source (*z* = 2.82; *p* = .800). For both gnammas and cattle troughs, mean vertebrate taxon richness per sample was greatest for filtered samples, followed by swept and sediment samples. None of the curves approached an asymptote by the fifth replicate but the gradients in curves from cattle trough samples were lower at the fifth replicate than were those from gnamma samples (Figure 3).

## 4 DISCUSSION

We detected a variety of mammals, birds, and amphibians from both artificial and natural semi‐permanent freshwater sources in the semi‐arid Great Western Woodlands, Western Australia. Our results indicate that sediment samples, filtered water samples and membranes swept through the water are all appropriate sampling approaches for detecting vertebrates in waterbodies in a semi‐arid environment using eDNA metabarcoding. However, the overall community structure detected varied both with the type of water source and the sampling technique. We observed that five replicates per water source was insufficient to detect all species interacting with these water sources. These findings are consistent with previous studies that have assessed the impact of sample collection on eDNA‐based terrestrial biodiversity assessment (e.g., Newton et al., 2022; van der Heyde et al., 2020) and demonstrate that this approach is most effective when replicate samples are collected from multiple water sources using a variety of sampling methods and substrates.

We detected only a relatively small proportion (10.2% of mammals, 6.5% of birds, and 7.15% of amphibians) of the vertebrate diversity recorded from the Great Western Woodland region (Department of Parks and Wildlife, 2013) but we did detect numerous threatened and elusive taxa (e.g., *Cinclosoma clarum*, *Petrogale lateralis*, *Northiella haematogaster*), as well as feral species (36.4% of total feral diversity in GWW) that threaten native biota. These results indicate that eDNA metabarcoding from water sources can contribute to biomonitoring in semi‐arid lands, particularly if sample collection involves numerous replicate samples, multiple sampling sources and techniques, and occurs over large spatial scales.

Overall, we detected more vertebrate taxa in samples from artificial water bodies than from natural water bodies (Figure 1 and Figure 2), despite them being found in similar habitats. Differences in accessibility of gnammas and cattle troughs for fauna may account for some of the variation in vertebrate richness and assemblage or that animals may preferentially use certain water bodies. For example, gnammas may present an injury risk with an uneven, slippery surface around the perimeter (Bayly, 1999) and the relative lack of plant cover on outcrops may result in greater vulnerability to predation while drinking for some species (Crosmary et al., 2012; Votto et al., 2022). Terrestrial mammals may therefore prefer to drink from above‐ground cattle troughs where the water surface is higher, reducing the risk of falling, and facilitating vigilance. This may be reflected by the greater diversity of mammals we detected from troughs. In contrast, we detected a greater diversity of birds from gnammas than from troughs (Figure 2). It is possible that diurnal birds may avoid cattle troughs due to the prevalence of large mammals, including predators (Fontaine et al., 2006) and humans (Meager et al., 2012) at these artificial water sources, especially since access is less of an issue for flighted birds.

Differences in ambient conditions in artificial and natural water sources related to shape and volume (e.g., diameter, depth, surface area, and edge complexity) may also influence persistence of vertebrate eDNA. For instance, ultraviolet exposure can increase eDNA degradation (Green et al., 2011 but see Bae & Wuertz, 2009) and high temperature denatures DNA molecules both directly and by enhancing microbial activity and enzyme kinetics (Fu et al., 2012; Okabe & Shimazu, 2007). Ledges, overhangs, and a smaller surface to volume ratio may provide protection from the impacts of temperature and ultraviolet for eDNA in gnammas compared to troughs, improving detection of small species which may deposit less eDNA, for example, birds compared to mammals. Our observations are consistent with previous studies that compare detection of terrestrial vertebrate taxa between multiple eDNA sources (Newton et al., 2022; van der Heyde et al., 2020) and support the conclusion that, when aiming to characterize arid or semi‐arid vertebrate biodiversity using eDNA metabarcoding, samples should be collected from both natural and artificial water sources to maximize the taxa detected. It is worth noting also that while eDNA is often proposed as a replacement of conventional methods, concomitant use of technology such as camera traps or conventional survey methods will value‐add to eDNA data (Newton et al., 2023; Ryan et al., 2022), particularly with regard to behavior of taxa near water holes, for example, just drinking or also bathing in the source (Harper, Handley, et al., 2019; Mas‐Carrió et al., 2021).

Sampling protocol can have a major impact on the biological community detected with eDNA metabarcoding (e.g., Stoeckle et al., 2017). Our results for vertebrate assemblages in a semi‐arid habitat support this finding. Sediment samples yielded significantly lower vertebrate taxonomic richness (50% of total richness) than did swept (88% of total richness) and filtered (84% of total richness) water samples (Figure 1); no unique taxa were detected in sediment samples while 13 taxa were only detected in water (swept and filtered) samples (Table 1 and Figure 2). Our findings are consistent with a previous study comparing eDNA detection in water and sediments for a single species, the great crested newt (*Triturus cristatus*; Buxton et al., 2018), but contradict some previous studies comparing eDNA detection in water and sediments (including those from arid lands; Palacios et al., 2020), which found that sampling sediments yielded higher biodiversity than water samples (Palacios et al., 2020; Turner et al., 2015). Environmental DNA can degrade rapidly in water, and the role of sediments in slowing eDNA degradation is well documented (Barnes et al., 2014; Hou et al., 2014; Romanowski et al., 1991). However, a variety of abiotic and biotic factors determine the rate of eDNA degradation and eDNA can be detectable in water for periods of only days to as long as weeks (Barnes et al., 2014) depending on environmental conditions. Our data suggest that either the rates of eDNA degradation in the water at our two sites were low, or, that the species we detected visited the water frequently enough to contribute detectable levels of eDNA still suspended in the water when sampling occurred.

To our knowledge, this is the first successful trial of swept eDNA collection from terrestrial water sources. Filtration through a membrane using a pump is the most common aquatic eDNA sampling method, typically processing large volumes of water rapidly. This approach is considered essential for accurately detecting the breadth of taxa present (Shu et al., 2020) but can be time consuming if the water contains particulates and/or organics. Our findings indicate that comparable taxonomic richness can be detected from filtered samples and swept samples from small semi‐arid habitat water sources (Figure 2). Similar findings were reported for fish detection in marine systems, where sample membranes were submerged for 4–24 h (Bessey et al., 2021). Interestingly, we only submerged the membranes for 15 s but still detected considerable faunal diversity. High concentrations of eDNA in isolated water bodies (Buxton et al., 2017) could account for the high vertebrate richness detected by eDNA metabarcoding of water samples collected by sweeping. To better understand the efficacy of sweeping we recommend future studies compare sweeping to filter volumes of 50 mL, 500 mL, and 1000 mL to address whether our results demonstrate effective sampling of different volumes with filtration and sweeping. We recognize that the 50 mL of filtered water we sampled here is considerably less than for most sampling protocols (Hunter et al., 2019 but see Day et al., 2019), but collecting small sample volumes was necessary due to the small volumes present at many of our study sites, and our results confirm that is enough for high eDNA concentration water bodies like the ones in this study. Additionally, the target substrate should also be considered since DNA in open water is more readily recovered using the current methods compared to DNA in sediment bound to clay particles, which requires a targeted approach (Sakata et al., 2020).

Our finding of comparable taxonomic richness between swept and filtered sampling approaches has profound implications for sampling turbid water, sampling in remote regions and for the logistics of collecting and transporting large volumes of samples. Optimal water filtering procedures are among the most important considerations when collecting and filtering turbid water samples. Particulates block water pumps, increasing the time it takes to filter samples and reducing the total number of samples that can be processed (Egeter et al., 2018; Hunter et al., 2019; Klymus et al., 2017; Turner et al., 2014). This issue is eliminated with sweeping. Membranes swept in water can also facilitate effective sample collection in remote regions where transporting pumps and/or large volumes of water samples and sourcing power to run pumps or freeze large volumes of water can be logistically challenging.

The application of eDNA metabarcoding as a biomonitoring tool to assess biological richness in terrestrial environments is becoming more frequent in the literature (e.g., van der Heyde et al., 2022) and commercially (e.g., Gold et al., 2021). However, the level of replication required to ensure that richness is not underestimated remains largely unexplored. Other studies assessing biological richness from arid zone water sources have used 1–12 biological replicates per environmental sample (e.g., Egeter et al., 2018; Furlan et al., 2020; Palacios et al., 2020) with optimal replication varying with species and context (Ficetola et al., 2015). Here we have demonstrated that five biological replicates collected from isolated water sources are insufficient to detect the true vertebrate richness at our study site (Figure 3). Even for the best‐performing water samples, rarefaction curves representing the accumulation of vertebrate richness with increasing replication effort continued to increase beyond the fifth replicate, particularly for samples collected from gnammas (Figure 3). Increasing replication reduces false‐negative results but may increase the probability of false positives stemming from contamination and inflates workload and analysis costs (Ficetola et al., 2015; Furlan et al., 2020). A need for high sample numbers for eDNA metabarcoding reduces the relative benefits of this approach as a rapid, cost‐effective biomonitoring tool compared to more conventional approaches (Ficetola et al., 2015; Furlan et al., 2020).

## 5 CONCLUSIONS

Our findings indicate that eDNA‐based monitoring of vertebrates in a semi‐arid habitat was most effective when samples were collected from both natural and artificial water sources using multiple sampling methods. We demonstrated that the water source significantly influenced determination of taxonomic richness and assemblage and that sediment samples had significantly less utility for determining vertebrate richness and were not associated with the identification of unique taxa compared to water samples either filtered or swept. We recommend that future studies should explore the use of sample storage buffers and eDNA extraction protocols that are tailored to reduce inhibition, especially in sediment samples. In contrast, we detected no difference in vertebrate richness between sweeping and filtering approaches for water samples. This finding has profound implications for the application of eDNA methods to inland regions and water sources, especially in remote areas, as sweep sampling dramatically reduces the time and logistics required to process samples. Finally, our observations demonstrated that increasing replication increased the number of vertebrate taxa detected, but >5 replicate samples are required to accurately sample small inland water bodies. In conclusion, eDNA metabarcoding for biomonitoring holds promise for applications in semi‐arid and arid lands but the design and execution of these studies must be carefully considered to overcome the unique limitations of this method in these environments.

## AUTHOR CONTRIBUTIONS

**Rupert Mcdonald:** Conceptualization (equal); data curation (equal); formal analysis (equal); investigation (equal); methodology (equal); writing – original draft (equal); writing – review and editing (equal). **Bill Bateman:** Conceptualization (equal); investigation (equal); writing – original draft (equal); writing – review and editing (equal). **Christine Cooper:** Conceptualization (equal); formal analysis (equal); investigation (equal); visualization (equal); writing – original draft (equal); writing – review and editing (equal). **Mieke Van Der Heyde:** Conceptualization (equal); data curation (equal); formal analysis (equal); investigation (equal); methodology (equal); supervision (equal); visualization (equal); writing – original draft (equal); writing – review and editing (equal). **Mousavi‐Derazmahalleh Mahsa:** Data curation (supporting); formal analysis (supporting). **Brock Hedges:** Investigation (supporting); writing – original draft (supporting); writing – review and editing (supporting). **Michelle Guzik:** Investigation (supporting); writing – original draft (supporting); writing – review and editing (supporting). **Paul Nevill:** Conceptualization (equal); funding acquisition (lead); investigation (equal); methodology (supporting); project administration (lead); supervision (lead); writing – original draft (equal); writing – review and editing (equal).

## FUNDING INFORMATION

This work was funded by IGO Limited and supported by the Pawsey Supercomputing Centre.

## Data Availability

Sequencing data and dada2 script are available at the Dryad Digital https://doi.org/10.5061/dryad.wdbrv15rb.

## References

[ece310014-bib-0001] Aird, D. , Ross, M. G. , Chen, W. S. , Danielsson, M. , Fennell, T. , Russ, C. , Jaffe, D. B. , Nusbaum, C. , & Gnirke, A. (2011). Analyzing and minimizing PCR amplification bias in Illumina sequencing libraries. Genome Biology, 12, R18. 10.1186/gb-2011-12-2-r18 21338519 PMC3188800

[ece310014-bib-0002] Altschul, S. F. , Gish, W. , Miller, W. , Myers, E. W. , & Lipman, D. J. (1990). Basic local alignment search tool. Molecular Ecology, 215, 403–410. 10.1016/S0022-2836(05)80360-2 2231712

[ece310014-bib-0003] Andrews, S. (2010). FastQC: A quality control tool for high throughput sequence data. Babraham Bioinformatics, Babraham Institute.

[ece310014-bib-0004] Bae, S. , & Wuertz, S. (2009). Rapid decay of host‐specific fecal Bacteroidales cells in seawater as measured by quantitative PCR with propidium monoazide. Water Research, 43, 4850–4859. 10.1016/j.watres.2009.06.053 19656546

[ece310014-bib-0005] Barnes, M. A. , & Turner, C. R. (2016). The ecology of environmental DNA and implications for conservation genetics. Conservation Genetics, 17, 1–17. 10.1007/s10592-015-0775-4

[ece310014-bib-0006] Barnes, M. A. , Turner, C. R. , Jerde, C. L. , Renshaw, M. A. , Chadderton, W. L. , & Lodge, D. M. (2014). Environmental conditions influence eDNA persistence in aquatic systems. Environmental Science & Technology, 48, 1819–1827. 10.1021/es404734p 24422450

[ece310014-bib-0007] Bayly, I. A. E. (1999). Review of how indigenous people managed for water in desert regions of Australia. Journal of the Royal Society of Western Australia, 82, 17–25.

[ece310014-bib-0008] Bessey, C. , Jarman, S. N. , Simpson, T. , Miller, H. , Stewart, T. , Keesing, J. K. , & Berry, O. (2021). Passive eDNA collection enhances aquatic biodiversity analysis. Communications biology, 4, 236. 10.1038/s42003-021-01760-8 33619330 PMC7900116

[ece310014-bib-0009] Boyer, F. , Mercier, C. , Bonin, A. , Le Bras, Y. , Taberlet, P. , & Coissac, E. (2016). Obitools: A unix‐inspired software package for DNA metabarcoding. Molecular Ecology Resources, 16, 176–182. 10.1111/1755-0998.12428 25959493

[ece310014-bib-0010] Bradford, T. , Adams, M. , Humphreys, W. F. , Austin, A. D. , & Cooper, S. J. B. (2010). DNA barcoding of stygofauna uncovers cryptic amphipod diversity in a calcrete aquifer in Western Australia's arid zone. Molecular Ecology Resources, 10, 41–50. 10.1111/j.1755-0998.2009.02706.x 21564989

[ece310014-bib-0011] Buxton, A. S. , Groombridge, J. J. , & Griffiths, R. A. (2017). Is the detection of aquatic environmental DNA influenced by substrate type? PLoS One, 12, e0183371. 10.1371/journal.pone.0183371 28813525 PMC5558973

[ece310014-bib-0012] Buxton, A. S. , Groombridge, J. J. , & Griffiths, R. A. (2018). Seasonal variation in environmental DNA detection in sediment and water samples. PLoS One, 13, e0191737. 10.1371/journal.pone.0191737 29352294 PMC5774844

[ece310014-bib-0013] Campbell, S. P. , Clark, J. A. , Crampton, L. H. , Guerry, A. D. , Hatch, L. T. , Hosseini, P. R. , & O'Connor, R. J. (2002). An assessment of monitoring efforts in endangered species recovery plans. Ecological Applications, 12, 674–681.

[ece310014-bib-0014] Carrasco‐Puga, G. , Díaz, F. P. , Soto, D. C. , Hernández‐Castro, C. , Conteras‐López, O. , Maldonado, A. , Latorre, C. , & Gutierrez, R. A. (2021). Revealing hidden plant diversity in arid environments. Ecography, 44, 98–111. 10.1111/ecog.05100

[ece310014-bib-0015] Cayuela, L. , Gotelli, N. J. , & Colwell, R. K. (2015). Ecological and biogeographic null hypotheses for comparing rarefaction curves. Ecological Monographs, 85, 437–455. 10.1890/14-1261.1

[ece310014-bib-0016] Chiarucci, A. , Bacaro, G. , Rocchini, D. , & Fattorini, L. (2008). Discovering and rediscovering the sample‐based rarefaction formula in the ecological literature. Community Ecology, 9, 121–123. 10.1556/ComEc.9.2008.1.14

[ece310014-bib-0017] Clarke, K. R. , & Gorley, R. N. (2015). PRIMER v7 (Version 7). PRIMER‐E.

[ece310014-bib-0018] Crosmary, W. G. , Makumbe, P. , Côte, S. D. , & Fritz, H. (2012). Vulnerability to predation and water constraints limit behavioural adjustments of ungulates in response to hunting risk. Animal Behaviour, 83, 1367–1376. 10.1016/j.anbehav.2012.03.004

[ece310014-bib-0019] Cross, S. L. , Craig, M. D. , Tomlinson, S. , Dixon, K. W. , & Bateman, P. W. (2020). Using monitors to monitor ecological restoration: Presence may not indicate persistence. Austral Ecology, 45, 921–932. 10.1111/aec.12905

[ece310014-bib-0020] Davies, S. J. J. F. (1972). Results of 40 Hours' continuous watch at five waterpoints in an Australian Desert. Emu, 72, 8–12. 10.1071/MU972008

[ece310014-bib-0021] Day, K. , Campbell, H. , Fisher, A. , Gibb, K. , Hill, B. , Rose, A. , & Jarman, S. N. (2019). Development and validation of an environmental DNA test for the endangered Gouldian finch. Endangered Species Research, 40, 171–182.

[ece310014-bib-0022] Deagle, B. E. , Jarman, S. N. , Coissac, E. , Pompanon, F. , & Taberlet, P. (2014). DNA metabarcoding and the cytochrome c oxidase subunit I marker: Not a perfect match. Biology Letters, 10, 20140562. 10.1098/rsbl.2014.0562 25209199 PMC4190964

[ece310014-bib-0023] Department of Parks and Wildlife . (2013). Great Western Woodlands: Draft strategic weed and feral animal management plan .

[ece310014-bib-0024] Durant, S. M. , Pettorelli, N. , Bashir, S. , Woodroffe, R. , Wacher, T. , Ornellas, D. , Ransom, C. , Abáigar, T. , Abdelgadir, M. , El Alqamy, H. , Beddiaf, M. , Belbachir, F. , Belbachir‐Bazi, A. , Berbash, A. A. , Beudels‐Jamar, R. , Boitani, L. , Breitenmoser, C. , Cano, M. , Chardonnet, P. , … Baillie, J. E. (2012). Forgotten biodiversity in desert ecosystems. Science, 336, 1379–1380. 10.1126/science.336.6087.1379 22700901

[ece310014-bib-0025] Edgar, R. (2010). Usearch: Sequencing, finishing, Analysis in the Future. Santa Fe.

[ece310014-bib-0026] Egeter, B. , Peixto, S. , Brito, J. C. , Jarman, S. , Puppo, P. , & Velo‐Antón, G. (2018). Challenges for assessing vertebrate diversity in turbid Saharan water‐bodies using environmental DNA. Genome, 61, 807–814. 10.1139/gen-2018-0071 30312548

[ece310014-bib-0027] Einoder, L. D. , Southwell, D. M. , Lahoz‐Monfort, J. J. , Gillespie, G. R. , Fisher, A. , & Wintle, B. A. (2018). Occupancy and detectability modelling of vertebrates in northern Australia using multiple sampling methods. PLoS One, 13, e0203304. 10.1371/journal.pone.0203304 30248104 PMC6152866

[ece310014-bib-0028] Epanchin‐Niell, R. S. , Haight, R. G. , Berec, L. , Kean, J. M. , & Liebhold, A. M. (2012). Optimal surveillance and eradication of invasive species in heterogeneous landscapes. Ecology Letters, 15, 803–812. 10.1111/j.1461-0248.2012.01800.x 22642613

[ece310014-bib-0029] Fahner, N. A. , Shokralla, S. , Baird, D. J. , & Hajibabaei, M. (2016). Large‐ scale monitoring of plants through environmental DNA metabar‐ coding of soil: Recovery, resolution, and annotation of four DNA markers. PLoS One, 11, 1–16. 10.1371/journal.pone.0157505 PMC4911152 27310720

[ece310014-bib-0030] Farrell, M. J. , Govender, D. , Hajibabaei, M. , van der Bank, M , & Davies, T. J. (2022). Environmental DNA as a management tool for tracking artificial waterhole use in savanna ecosystems. Biological Conservation, 274, 109712. 10.1016/j.biocon.2022.109712

[ece310014-bib-0031] Fernandes, K. , van der Heyde, M. , Bunce, M. , Dixon, K. , Harris, R. J. , Wardell‐Johnson, G. , & Nevill, P. G. (2018). DNA metabarcoding—A new approach to fauna monitoring in mine site restoration. Restoration Ecology, 26, 1098–1107. 10.1111/rec.12868

[ece310014-bib-0032] Ficetola, G. F. , Pansu, J. , Bonin, A. , Coissac, E. , Giguet‐Covex, C. , De Barba, M. , Gielly, L. , Lopes, C. M. , Boyer, F. , Pompanon, F. , Rayé, G. , & Taberlet, P. (2015). Replication levels, false presences, and the estimation of presence/absence from eDNA metabarcod‐ ing data. Molecular Ecology Resources, 15, 543–556. 10.1111/1755-0998.12338 25327646

[ece310014-bib-0033] Fontaine, J. J. , Martin, T. E. , Coulson, T. , & Losos, J. B. (2006). Habitat selection responses of parents to offspring predation risk: An experimental test. The American Naturalist, 168, 811–818. 10.1086/508297 17109323

[ece310014-bib-0034] Fox, E. , McNee, S. , & Douglas, T. (2016). Birds of the great Western woodlands: Report for the nature conservancy. BirdLife Australia.

[ece310014-bib-0036] Fu, X. H. , Wang, L. , Le, Y. Q. , & Hu, J. J. (2012). Persistence and renaturation efficiency of thermally treated waste recombinant DNA in defined aquatic microcosms. Journal of Environmental Science and Health, 47, 1975–1983. 10.1080/10934529.2012.695260 22870994

[ece310014-bib-0037] Furlan, E. M. , Davis, J. , & Duncan, R. P. (2020). Identifying error and accurately interpreting environmental DNA metabarcoding results: A case study to detect vertebrates at arid zone waterholes. Molecular Ecology Resources, 20, 1259–1276. 10.1111/1755-0998.13170 32310337

[ece310014-bib-0038] Gold, Z. , Sprague, J. , Kushner, D. J. , Zerecero Marin, E. , & Barber, P. H. (2021). eDNA metabarcoding as a biomonitoring tool for marine protected areas. PLoS One, 16, e0238557. 10.1371/journal.pone.0238557 33626067 PMC7904164

[ece310014-bib-0039] Green, H. C. , Shanks, O. C. , Sivaganesan, M. , Haugland, R. A. , & Field, K. G. (2011). Differential decay of human faecal Bacteroides in marine and freshwater. Environmental Microbiology, 13, 3235–3249. 10.1111/j.1462-2920.2011.02549.x 21883797

[ece310014-bib-0040] Harper, L. H. , Handley, L. L. , Carpenter, A. I. , Ghazali, M. , Muri, C. D. , Macgregor, C. J. , Logan, T. W. , Law, A. , Breithaupt, T. , Read, D. S. , McDevitt, A. D. , & Hänfling, B. (2019). Environmental DNA (eDNA) metabarcoding of pond water as a tool to survey conservation and management priority mammals. Biological Conservation, 238, 1–11. 10.1016/j.biocon.2019.108225

[ece310014-bib-0041] Harper, L. R. , Buxton, A. S. , Rees, H. C. , Bruce, K. , Brys, R. , Halfmaerten, D. , & Hänfling, B. (2019). Prospects and challenges of environmental DNA (eDNA) monitoring in freshwater ponds. Hydrobiologia, 825, 25–41. 10.1007/s10750-018-3750-5

[ece310014-bib-0042] Harrison, J. B. , Sunday, J. M. , & Rogers, S. M. (2019). Predicting the fate of eDNA in the environment and implications for studying biodiversity. Proceedings of the Biological Sciences, 286, 20191409. 10.1098/rspb.2019.1409 PMC6892050 31744434

[ece310014-bib-0043] Hedges, B. A. , Austin, A. D. , Conran, J. G. , Taylor, G. S. , Madden, C. P. , & Weinstein, P. (2021). A likely association of damselflies with the habitat heterogeneity provided by the freshwater swamp lily, *Ottelia ovalifolia*, in Eyre peninsula granite rock‐holes, with a review of potential threats to this ephemeral habitat. Transactions of the Royal Society of South Australia, 145, 152–167. 10.1080/03721426.2021.1996878

[ece310014-bib-0044] Herrick, J. E. , Schuman, G. E. , & Rango, A. (2006). Monitoring ecological processes for restoration projects. Journal for Nature Conservation, 14, 161–171. 10.1016/j.jnc.2006.05.001

[ece310014-bib-0045] Hou, Y. , Wu, P. , & Zhu, N. (2014). The protective effect of clay minerals against damage to adsorbed DNA induced by cadmium and mercury. Chemosphere, 95, 206–212. 10.1016/j.chemosphere.2013.08.069 24047649

[ece310014-bib-0046] Hunter, M. E. , Ferrante, J. A. , Meigs‐Freimd, G. , & Ulmer, A. (2019). Improving eDNA yield and inhibitor reduction through increased water volumes and multi‐filter isolation techniques. Scientific Reports, 9, 5259. 10.1038/s41598-019-40977-w1 30918268 PMC6437164

[ece310014-bib-0047] James, C. D. , Landsberg, J. , & Morton, S. R. (1999). Provision of watering points in the Australian arid zone: A review of effects on biota. Journal of Arid Environments, 41, 87–121. 10.1006/jare.1998.0467

[ece310014-bib-0048] James, J. J. , Sheley, R. L. , Erickson, T. , Rollins, K. S. , Taylor, M. H. , & Dixon, K. W. (2013). A systems approach to restoring degraded drylands. Journal of Applied Ecology, 50, 730–739. 10.1111/1365-2664.12090

[ece310014-bib-0049] Ji, Y. , Ashton, L. , Pedley, S. M. , Edwards, D. P. , Tang, Y. , Nakamura, A. , Kitching, R. , Dolman, P. M. , Woodcock, P. , Edwards, F. A. , Larsen, T. H. , Hsu, W. W. , Benedick, S. , Hamer, K. C. , Wilcove, D. S. , Bruce, C. , Wang, X. , Levi, T. , Lott, M. , & Yu, D. W. (2013). Reliable, verifiable and efficient monitoring of biodiversity via metabarcoding. Ecology Letters, 16, 1245–1257. 10.1111/ele.12162 23910579

[ece310014-bib-0050] Kelly, R. P. , Port, J. A. , Yamahara, K. M. , Martone, R. G. , Lowell, N. , Thomsen, P. F. , Mach, M. E. , Bennet, M. , Prahler, E. , Caldwell, M. R. , & Crowder, L. B. (2014). Harnessing DNA to improve environmental management: Genetic monitoring can help public agencies implement environmental laws. Science, 344, 1455–1456. http://www.jstor.org/stable/24744755 24970068 10.1126/science.1251156

[ece310014-bib-0051] Kindt, R. , & Coe, R. (2005). Tree diversity analysis: A manual and software for common statistical methods for ecological and biodiversity studies. In World agroforestry Centre (ICRAF). Nairobi.

[ece310014-bib-0052] Kitano, T. , Umetsu, K. , Tian, W. , & Osawa, M. (2007). Two universal primer sets for species identification among vertebrates. International Journal of Legal Medicine, 121, 423–427. 10.1007/s00414-006-0113-y 16845543

[ece310014-bib-0053] Klymus, K. E. , Richter, C. A. , Thompson, N. , & Hinck, J. E. (2017). Metabarcoding of environmental DNA samples to explore the use of uranium mine containment ponds as a water source for wildlife. Diversity, 9, 54. 10.3390/d9040054

[ece310014-bib-0054] Knezevic, S. Z. , Streibig, J. C. , & Ritz, C. (2007). Utilizing R software package for dose‐response studies: The concept and data analysis. Weed Technology, 21, 840–848. 10.1614/WT-06-161.1

[ece310014-bib-0055] Korine, C. , Adams, R. , Russo, D. , Fisher‐Phelps, M. , & Jacobs, D. (2016). Bats and water: Anthropogenic alterations threaten global bat populations. In Bats in the Anthropocene: Conservation of bats in a changing world (pp. 215–241). Springer.

[ece310014-bib-0056] Lemons, J. , & Victor, R. (2003). Case studies on conserving and sustainably using biodiversity in arid and semiarid regions of southern nations. In J. Lemons , R. Victor , & D. Schaffer (Eds.), Conserving biodiversity in arid regions: Best practices in developing nations (1st ed., pp. 1–26). Kluwer Academic Publishers. 10.1007/978-1-4615-0375-0

[ece310014-bib-0057] Letnic, M. , Webb, J. K. , Jessop, T. S. , Florance, D. , & Dempster, T. (2014). Artificial water points facilitate the spread of an invasive vertebrate in arid Australia. Journal of Applied Ecology, 51, 795–803.

[ece310014-bib-0058] Mas‐Carrió, E. , Schneider, J. , Nasanbat, B. , Ravchig, S. , Buxton, M. , Nyamukondiwa, C. , Stoffel, C. , Augugliaro, C. , Ceacero, F. , Taberlet, P. , Glaizot, O. , Christie, P. , & Fumagalli, L. (2021). Assessing environmental DNA metabarcoding and camera trap surveys as complementary tools for biomonitoring of remote desert water bodies. Environmental DNA, 5, 1–16. 10.1002/edn3.274

[ece310014-bib-0059] Meager, J. J. , Schlacher, T. A. , & Nielsen, T. (2012). Humans alter habitat selection of birds on ocean‐exposed sandy beaches. Diversity and Distributions, 18, 294–306. 10.1111/j.1472-4642.2011.00873.x

[ece310014-bib-0060] Mousavi‐Derazmahalleh, M. , Stott, A. , Lines, R. , Peverley, G. , Nester, G. , Simpson, T. , Zawierta, M. , La Pierre, D. , Bunce, M. , & Christophersen, C. T. (2021). eDNAFlow, an automated, reproducible and scalable workflow for analysis of environmental DNA (eDNA) sequences exploiting Nextflow and singularity. Molecular Ecology Resources, 21, 1697–1704. 10.1111/1755-0998.13356 33580619

[ece310014-bib-0061] Murray, D. C. , Coghlan, M. L. , & Bunce, M. (2015). From benchtop to desktop: Important considerations when designing amplicon sequencing workflows. PLoS One, 10, e0124671. 10.1371/journal.pone.0124671 25902146 PMC4406758

[ece310014-bib-0062] Newbey, K. R. , Dell, J. , How, R. A. , & Hnatiuk, R. J. (1984). The biological survey of the eastern goldfields of Western Australia: Part 2 Widgiemooltha – Zanthus study area. Western Australian Museum.

[ece310014-bib-0063] Newton, J. P. , Bateman, P. W. , Heydenrych, M. J. , Kestel, J. H. , Dixon, K. W. , Prendergast, K. S. , White, N. E. , & Nevill, P. (2023). Monitoring the birds and the bees: Environmental DNA metabarcoding of flowers detects plant–animal interactions. Environmental DNA, 5, 1–15. 10.1002/edn3.399

[ece310014-bib-0064] Newton, J. P. , Bateman, P. W. , Heydenrych, M. J. , Mousavi‐Derazmahalleh, M. , & Nevill, P. (2022). Home is where the hollow is: Revealing vertebrate tree hollow user biodiversity with eDNA metabarcoding. Environmental DNA, 4(5), 1078–1091.

[ece310014-bib-0065] O'Farrell, P. J. , Reyers, B. , Le Maitre, D. C. , Milton, S. J. , Egoh, B. , Maherry, A. , Colvin, C. , Atkinson, D. , De Lange, W. , Blignaut, J. N. , & Cowling, R. M. (2010). Multi‐functional landscapes in semi‐arid environments: Implications for biodiversity and ecosystem services. Landscape Ecology, 25, 1231–1246. 10.1007/s10980-010-9495-9

[ece310014-bib-0066] Okabe, S. , & Shimazu, Y. (2007). Persistence of host‐specific Bacteroides‐ Prevotella 16S rRNA genetic markers in environmental waters: Effects of temperature and salinity. Applied Microbiology and Biotechnology, 76, 935–944. 10.1007/s00253-007-1048-z 17598108

[ece310014-bib-0067] Palacios, M. M. , Curd, E. , Edalati, K. , Renshaw, M. A. , Dunn, R. , Potter, D. , Fraga, N. , Moore, J. , Saiz, J. , Wayne, R. , & Parker, S. S. (2020). The utility of environmental DNA from sediment and water samples for recovery of observed plant and animal species from four Mojave Desert springs. Environmental DNA, 3, 214–230. 10.1002/edn3.161

[ece310014-bib-0068] R Core Team . (2018). R: A language and environment for statistical computing. R Foundation for Statistical Computing. https://www.R‐project.org

[ece310014-bib-0069] Razgour, O. , Persey, M. , Shamir, U. , & Korine, C. (2018). The role of climate, water and biotic interactions in shaping biodiversity patterns in arid environments across spatial scales. Diversity and Distributions, 24, 1440–1452. 10.1111/ddi.12773

[ece310014-bib-0070] Redfern, J. V. , Grant, R. , Biggs, H. , & Getz, W. M. (2003). Surface‐water constraints on herbivore foraging in the Kruger National Park, South Africa. Ecology, 84, 2092–2107. 10.1890/01-0625

[ece310014-bib-0071] Riaz, T. , Shehzad, W. , Viari, A. , Pompanon, F. , Taberlet, P. , & Coissac, E. (2011). EcoPrimers: Inference of new DNA barcode markers from whole genome sequence analysis. Nucleic Acids Research, 39, 1–11. 10.1093/nar/gkr732 21930509 PMC3241669

[ece310014-bib-0073] Rodgers, T. W. , & Mock, M. E. (2015). Drinking water as a source of environmental DNA for the detection of terrestrial wildlife species. Conservation Genetics Resources, 7, 693–696. 10.1007/s12686-015-0478-7

[ece310014-bib-0074] Romanowski, G. , Lorenz, M. G. , & Wackernagel, W. (1991). Adsorption of plasmid DNA to mineral surfaces and protection against DNase I. Applied and Environmental Microbiology, 57, 1057–1061. 10.1128/aem.57.4.1057-1061.1991 1647748 PMC182845

[ece310014-bib-0075] Ryan, E. , Bateman, P. , Fernandes, K. , van der Heyde, M. , & Nevill, P. (2022). eDNA metabarcoding of log hollow sediments and soils highlights the importance of substrate type, frequency of sampling and animal size, for vertebrate species detection. Environmental DNA, 4, 940–953. 10.1002/edn3.306

[ece310014-bib-0076] Sakata, M. K. , Yamamoto, S. , Gotoh, R. O. , Miya, M. , Yamanaka, H. , & Minamoto, T. (2020). Sedimentary eDNA provides different information on timescale and fish species composition compared with aqueous eDNA. Environmental. DNA, 2, 505–518. 10.1002/edn3.75

[ece310014-bib-0077] Sarri, C. , Stamatis, C. , Sarafidou, T. , Galara, I. , Godosopoulos, V. , Kolovos, M. , Liakou, C. , Tastsoglou, S. , & Mamuris, Z. (2014). A new set of 16s rRNA universal primers for identification of animal species. Food Control, 43, 35–41. 10.1016/j.foodcont.2014.02.036

[ece310014-bib-0078] Schneider, N. A. , & Griesser, M. (2009). Influence and value of different water regimes on avian species richness in arid inland Australia. Biodiversity and Conservation, 18, 457–471.

[ece310014-bib-0079] Schubert, M. , Lindgreen, S. , & Orlando, L. (2016). AdapterRemoval v2: Rapid adapter trimming, identification, and read merging. BMC Research Notes, 9, 88. 26868221 10.1186/s13104-016-1900-2 PMC4751634

[ece310014-bib-0080] Shu, L. , Ludwig, A. , & Peng, Z. (2020). Standards for methods utilizing environmental DNA for detection of fish species. Gene, 11, 296. 10.3390/genes11030296 PMC7140814 32168762

[ece310014-bib-0081] Staats, M. , Arulandhu, A. J. , Gravendeel, B. , Holst‐Jensen, A. , Scholtens, I. , Peelen, T. , Prins, T. W. , & Kok, E. (2016). Advances in DNA metabarcoding for food and wildlife forensic species identification. Analytical and Bioanalytical Chemistry, 408, 4615–4630. 10.1007/s00216-016-9595-8 27178552 PMC4909793

[ece310014-bib-0082] Stoeckle, B. C. , Beggel, S. , Cerwenka, A. F. , Motivans, E. , Kuehn, R. , & Geist, J. (2017). A systematic approach to evaluate the influence of environmental conditions on eDNA detection success in aquatic ecosystems. PLoS One, 12, e0189119. 10.1371/journal.pone.0189119 29220394 PMC5722286

[ece310014-bib-0083] Taberlet, P. , Coissac, E. , Pompanon, F. , Brochmann, C. , & Willerslev, E. (2012). Towards next‐generation biodiversity assessment using DNA metabarcoding. Molecular Ecology, 21, 2045–2050. 10.1111/j.1365-294X.2012.05470.x 22486824

[ece310014-bib-0084] Takahara, T. , Minamoto, T. , Yamanaka, H. , Doi, H. , & Kawabata, Z. (2012). Estimation of fish biomass using environmental DNA. PLoS One, 7, e35868. 10.1371/journal.pone.0035868 22563411 PMC3338542

[ece310014-bib-0085] Takahashi, M. , Saccò, M. , Kestel, J. H. , Nester, G. , Campbell, M. A. , Van Der Heyde, M. , & Allentoft, M. E. (2023). Aquatic environmental DNA: A review of the macro‐organismal biomonitoring revolution. Science of the Total Environment, 873, 162322. 36801404 10.1016/j.scitotenv.2023.162322

[ece310014-bib-0086] Taylor, P. G. (1996). Reproducibility of ancient DNA sequences from extinct Pleistocene fauna. Molecular Biology and Evolution, 13, 283–285. 10.1093/oxfordjournals.molbev.a025566 8583902

[ece310014-bib-0087] Thompson, K. A. , & Newmaster, S. G. (2014). Molecular taxonomic tools provide more accurate estimates of species richness at less cost than traditional morphology‐based taxonomic practices in a vegetation survey. Biodiversity and Conservation, 23, 1411–1424. 10.1007/s10531-014-0672-z

[ece310014-bib-0088] Thomsen, P. F. , Kielgast, J. , Iversen, L. L. , Wiuf, C. , Rasmussen, M. , Gilbert, M. T. P. , Orlando, L. , & Willerslev, E. (2012). Monitoring endangered freshwater biodiversity using environmental DNA. Molecular Ecology, 21, 2565–2573. 10.1111/j.1365-294X.2011.05418.x 22151771

[ece310014-bib-0089] Turner, C. R. , Miller, D. J. , Coyne, K. J. , & Corush, J. (2014). Improved methods for capture, extraction, and quantitative assay of environmental DNA from Asian bigheaded carp (*Hypophthalmichthys spp*). PLoS One, 9, e114329. 10.1371/journal.pone.0114329 25474207 PMC4256254

[ece310014-bib-0090] Turner, C. R. , Uy, K. L. , & Everhart, R. C. (2015). Fish environmental DNA is more concentrated in aquatic sediments than surface water. Biological Conservation, 183, 93–102. 10.1016/j.biocon.2014.11.017

[ece310014-bib-0091] Ushio, M. , Fukuda, H. , Inoue, T. , Makoto, K. , Kishida, O. , Sato, K. , Murata, K. , Nikaido, M. , Sado, T. , Sato, Y. , Takeshita, M. , Iwasaki, W. , Yamanaka, H. , Kondoh, M. , & Miya, M. (2017). Environmental DNA enables detection of terrestrial mammals from forest pond water. Molecular Ecology Resources, 17, 63–75. 10.1111/1755-0998.12690 28603873

[ece310014-bib-0092] Valentini, A. , Taberlet, P. , Miaud, C. , Civade, R. , Herder, J. , Thomsen, P. F. , Bellemain, E. , Besnard, A. , Coissac, E. , Boyer, F. , Gaboriaud, C. , Jean, P. , Poulet, N. , Roset, N. , Copp, G. H. , Geniez, P. , Pont, D. , Argillier, C. , Baudoin, J. , … Dejean, T. (2016). Next‐generation monitoring of aquatic biodiversity using environmental DNA metabarcoding. Molecular Ecology, 25, 929–942. 10.1111/mec.13428 26479867

[ece310014-bib-0093] van Der Heyde, M. , Bateman, P. W. , Bunce, M. , Wardell‐Johnson, G. , White, N. E. , & Nevill, P. (2021). Scat DNA provides important data for effective monitoring of mammal and bird biodiversity. Biodiversity and Conservation, 30, 3585–3602.

[ece310014-bib-0094] van der Heyde, M. , Bunce, M. , & Nevill, P. (2022). Key factors to consider in the use of environmental DNA metabarcoding to monitor terrestrial ecological restoration. Science of the Total Environment, 848, 157617. 10.1016/j.scitotenv.2022.157617 35901901

[ece310014-bib-0095] van der Heyde, M. E. , Bunce, M. , Wardell‐Johnson, G. , Fernandes, K. , White, N. E. , & Nevill, P. (2020). Testing multiple substrates for terrestrial biodiversity monitoring using environmental DNA metabarcoding. Molecular Ecology Resources, 20, 732–745. 10.1111/1755-0998.13148 32065512

[ece310014-bib-0096] Votto, S. E. , Schlesinger, C. , Dyer, F. , Caron, V. , & Davis, J. (2022). The role of fringing vegetation in supporting avian access to arid zone waterholes. Emu‐Austral Ornithology, 122, 1–15. 10.1080/01584197.2022.2041441

[ece310014-bib-0097] Wang, S. , Yan, Z. , Hänfling, B. , Zheng, X. , Wang, P. , Fan, J. , & Li, J. (2021). Methodology of fish eDNA and its applications in ecology and environment. The Science of the Total Environment, 755, 142622. 10.1016/j.scitotenv.2020.142622 33059148

[ece310014-bib-0098] Waudby, H. P. , Petit, S. , & Gill, M. J. (2019). The scientific, financial and ethical implications of three common wildlife‐trapping designs. Wildlife Research, 46, 690–700. 10.1071/WR19084

[ece310014-bib-0099] Wickham, H. , & Sievert, C. (2016). Ggplot2: Elegant graphics for data analysis (2nd ed.). Springer International Publishing AG. 10.1007/978-3-319-24277-4

[ece310014-bib-0100] Yu, D. W. , Ji, Y. , Emerson, B. C. , Wang, X. , Ye, C. , Yang, C. , & Ding, Z. (2012). Biodiversity soup: Metabarcoding of arthropods for rapid biodiversity assessment and biomonitoring. Methods in Ecology and Evolution, 3, 613–623. 10.1111/j.2041-210X.2012.00198.x

